# Heterogeneous development of children with Congenital Zika Syndrome-associated microcephaly

**DOI:** 10.1371/journal.pone.0256444

**Published:** 2021-09-15

**Authors:** Juan P. Aguilar Ticona, Nivison Nery, Simon Doss-Gollin, Claudia Gambrah, Millani Lessa, Valmir Rastely-Júnior, Adriana Matos, Bruno de Paula Freitas, Ana Borja, Elsio A. Wunder, Verena Ballalai, Carina Vieira, Jaqueline S. Cruz, Daiana de Oliveira, Danielle Bastos Araujo, Danielle B. Oliveira, Denicar Lina Nascimento Fabris Maeda, Erica A. Mendes, Camila Pereira Soares, Edison L. Durigon, Luis Carlos de Souza Ferreira, Rubens Belfort, Antonio R. P. Almeida, Jamary Oliveira-Filho, Mitermayer G. Reis, Albert I. Ko, Federico Costa

**Affiliations:** 1 Instituto de Saúde Coletiva, Universidade Federal da Bahia, Salvador, BA, Brazil; 2 Instituto Gonçalo Moniz, Fundação Oswaldo Cruz, Ministério da Saúde, Salvador, BA, Brazil; 3 Precision Vaccines Program, Division of Infectious Diseases, Boston Children’s Hospital, Boston, MA, United States of America; 4 Department of Epidemiology of Microbial Diseases, Yale School of Public Health, New Haven, Connecticut, United States of America; 5 Faculdade de Medicina da Bahia, Universidade Federal da Bahia, Salvador, BA, Brazil; 6 Hospital Geral Roberto Santos (HGRS), Salvador, BA, Brazil; 7 Faculdade de Medicina, Universidade Federal de São Paulo, São Paulo, SP, Brazil; 8 Departamento de Fonoaudiologia, Instituto de Ciências da Saúde, Universidade Federal da Bahia, Salvador, BA, Brazil; 9 Departamento de Microbiologia, Instituto de Ciências Biomédicas, Universidade de São Paulo, São Paulo, Brazil; 10 Laboratório de Desenvolvimento de Vacinas, Departamento de Microbiologia, Instituto de Ciências Biomédicas, Universidade de São Paulo, São Paulo, Brazil; 11 Programa de Pós-graduação em Ciências da Saúde (PPgCS), Universidade Federal da Bahia, Salvador, BA, Brazil; Amsterdam Universitair Medische Centra, NETHERLANDS

## Abstract

**Objective:**

To describe the neurological and neurodevelopmental outcomes of children with Congenital Zika Syndrome (CZS) associated microcephaly beyond 2 years of age.

**Method:**

We followed children with CZS-associated microcephaly in an outpatient clinic in Salvador, Brazil. Neurological and neurodevelopmental assessments were performed using the Hammersmith Infant Neurological Examination (HINE) and Bayley Scales of Infant and Toddler Neurodevelopment (Bayley-III) respectively.

**Results:**

Of the 42 children included, 19 were male (45.2%); median (interquartile range) age at neurological evaluation was 28 (25–32) months, and 36 (85.7%) had severe microcephaly. HINE and Bayley-III results were completed for 35/42 (83.3%) and 33/42 (78.5%) children respectively. Bayley-III identified a severe developmental delay in 32/33 (97.0%) children while 1/33 (3.0%) had only a mild delay. In the multivariable analysis, we found that Bayley-III and HINE scores were correlated. Better HINE scores were associated with higher Bayley-III cognitive raw scores (β = 0.29; CI 95% = 0.02–0.57) and motor raw scores (β = 0.43; CI 95% = 0.04–0.82) after adjusting for head circumference, prematurity, and age at neurodevelopmental evaluation. Furthermore, we found that greater head circumference at follow up was associated with higher cognitive (β = 1.27; CI 95% = 0.01–2.53) and motor raw scores (β = 2.03; CI 95% = 0.25–3.81).

**Conclusion:**

Children with CZS-associated microcephaly demonstrate severe neurodevelopmental delays and slower growth rates than their peers over time. Still, they have remarkably heterogeneous neurodevelopmental profiles according to neurological exam scores which correlate with their long-term outcomes. We found that HINE scores effectively captured the heterogeneity of neurological capabilities among these children and could be predictive of cognitive and motor development progress.

## Introduction

Congenital Zika Virus (ZIKV) infection is associated with severe neurological abnormalities such as microcephaly and central nervous system malformation [1]. Congenital Zika syndrome (CZS) refers to the presence of microcephaly or other congenital anomalies and alterations such as brain disruption sequence, ocular lesions, congenital contractures and neurodevelopmental impairments which have also been associated with congenital ZIKV infection [2, 3].

The clinical evolution and prognosis of children with CZS-associated microcephaly may vary significantly and has not yet been fully delineated after these children reach two years of age. Previous studies have found major neurodevelopmental delays of around 20 months in children less than 2 years of age with CZS-associated microcephaly, [4] although cases have also been identified in which children with CZS-associated microcephaly underwent approximately normal neurodevelopment [5–8], including one case report which describes a child showing normal neurodevelopment despite CZS-associated microcephaly (head circumference Z-score −2.4 SD) [9]. These heterogeneous findings suggest significant variation in developmental outcomes for children exposed to ZIKV *in utero*. Given that neurodevelopment is a dynamic process, it is important to track these children past two years of age in order to learn whether these differences persist over time. This heterogeneity has not been well described, which may, in part, be due to difficulties in effectively assessing developmental differences among children with severe disabilities [10]. Furthermore, there is little information about factors associated with this heterogeneity. This study aims to characterize the neurodevelopmental outcomes of children with CZS-associated microcephaly between 24–40 months of age.

## Materials and methods

### Study site and participants

We performed a prospective study of children from the Microcephaly Outpatient Clinic at the *Hospital Geral Roberto Santos* (HGRS) in Salvador, Bahia, Brazil. We enrolled children with a head circumference more than two standard deviations (<-2 SD) below average, according to the standards set by the International Fetal and Newborn Growth Consortium for the 21^st^ century (INTERGROWTH-21st), who were born during the peak of the ZIKV epidemic in Salvador between October 1^st^, 2015 and January 31th, 2016 at HGRS or nearby health centers and who received follow-up attention at the HGRS Microcephaly Outpatient Clinic.

A multidisciplinary team composed of physiotherapists, speech therapists, nurses, and physicians, including an ophthalmologist and a neurologist, collected information on mothers and their children based on interviews, medical records, and clinical evaluations. Sociodemographic statistics were obtained during the interviews at birth and at follow-up. All data were recorded and managed in REDCap (Research Electronic Data Capture).

### Intrauterine ZIKV exposure

Congenital ZIKV exposure was defined using a Plaque Reduction Neutralization Test (PRNT50) for maternal serum samples. These serum samples were collected either at the child’s birth or during the follow up evaluation. Serum samples were considered positive when anti-ZIKV neutralizing antibody titer was ≥ 1: 20 for ZIKV.

### Clinical and developmental outcomes

INTERGROWTH‐21st Fetal Growth Standards were used to evaluate children’s head circumferences (HC) at birth and to adjust for gestational age and sex [11]. This was used to define microcephaly (<-2 SD) as inclusion criteria for this study. World Health Organization (WHO) parameters were used to evaluate the child’s anthropometric growth during the follow up in the second year of life. The child’s length and weight were also measured and adjusted by age and sex [12].

We performed neurological evaluations using the Hammersmith Infant Neurological Examination (HINE) [12]. HINE is a scorable clinical neurological exam comprised of three sections. The first section is the main section, and is a neurological assessment which includes 26 items (score range between 0–78) to evaluate cranial nerve function, posture, movements, tone, and reflexes. The second section is comprised of 8 items (score range between 0–26) and evaluates motor function by age relative to standard milestones in childhood development. The third section is composed of three items and evaluates child behavior with regard to consciousness or alertness, emotional state, and social orientation, (score range between 3–15). In children over 18 months of age, an optimal HINE score for the first section is ≥74 [12]. In children with neurodevelopmental impairments, a score <40 was associated with severe motor impairment while scores between 41 and 60 were associated with less severe motor impairment [13]. Previous studies in the literature focus on the first section, and so although we performed all three components, our subsequent analysis focused on the first section while the others were only used in our initial characterizations of the children.

We also used the Bayley Scales of Infant and Toddler Development, 3rd edition (Bayley-III) to evaluate cognitive, language (expressive and receptive), and motor (fine and gross) development of children in our cohort[14]. Bayley-III is a neurodevelopmental tool for children between 16 days and 42 months of age. For each scale, raw scores, age-corrected scores, percentile scores, and composite scores were obtained. Children were classified based on their composite scores as having severely delayed (composite score ≤70 [≤-2 SD]), mildly delayed (71 to 85 [-2 SD to -1 SD]), or normal development (>85) [14–16].

Ophthalmologic evaluations were performed using standard techniques to identify and evaluate CZS-associated abnormalities. These included external ocular examination, functional ocular examinations, ocular biomicroscopy, and indirect ophthalmoscopy with pupillary dilation. The visual function assessment included the following items: visual acuity, sensitivity to light, ocular fixation, stability, object tracking, visual contact, social smile response, facial responses to visual stimuli, and facial expression imitation. They were evaluated by an ophthalmologist and a determination of inadequate visual function was defined as children with one or more abnormal responses during the assessment. Auditory evaluations included behavioral hearing screening, development of auditory skills and cochleo-palpebral reflex which were performed using an adapted conditioned play audiometry test (Simonek hearing kit), designed to screen children up to 48 months of age [16]. This test evaluates reflexes, attention, location, and orientation in front of different auditory stimuli using objects with different frequency levels (from 38.2 to 95.1 decibels). Furthermore, brainstem evoked response audiometry (BERA) and transient evoked otoacoustic emissions (TEOAE) tests were performed. Cranial computed tomography scan (CT) was obtained during the first year of life at the HGRS.

### Statistical analysis

Data analysis was performed using R Studio v3.6.1. Data were summarized using descriptive statistics. To evaluate the differences between head circumference Z-scores at birth and during the follow up, we used the paired samples Wilcoxon test. We used Spearman’s rank correlation to evaluate the relationships between Bayley scales, age at follow up, and HINE scores. In the multivariable analysis, we excluded children with incomplete neurological and neurodevelopmental evaluations. Stepwise linear regression analyses were performed to calculate the association between each independent variable and neurodevelopment (Bayley scales). Results were considered statistically significant at p < 0.05.

### Ethical aspects

The study was approved by the Institutional Review Boards of Yale University (1006006956) and the ethics committee of the Hospital Geral Roberto Santos–Bahia (1.866.918). Written informed consent was obtained from the parents of all studied patients.

## Results

We included 42 children in our study, 29 of whom were born at HGRS and 13 which were born at another health center but followed at the HGRS Microcephaly Outpatient Clinic. Among them, 19/42 (45.2%) were male and 4/42 (9.5%) were born prematurely (<37 weeks of gestation), 2/42 (4.5%) born with 33 and 34 weeks (moderate preterm) and 2/42 (4.5%) born with 36 weeks (late preterm) and 19/28 (67.9%) presented with postneonatal epilepsy (Table 1). Sociodemographic characteristics of the mothers are summarized in the S1 Table. Median maternal age at delivery was 25 years (range 15–42), 20/37 (54.1%) self-declared as black, and 11/36 (30.6%) reported having less than nine years of schooling. ZIKV exposure was positive in 40/40 (100%) cases with available positive PRNT result (Table 1). We were not able to collect blood sample of two children, so laboratory results were not available. However, those two children were included in the study and followed because they had clinical indications of CZS (microcephaly and severe neurodevelopmental delays), neuroimaging findings characteristic of CZS, and no serologic evidence of other congenital infections (TORCHS). CT scans were available for 39/42 (92.6%) participants. Among them, the most frequent findings were ventriculomegaly 34/39 (87.2%), parenchymal 33/39 (84.6%) and subcortical 30/39 (76.9%) calcification, and simplified gyral pattern 31/39 (79.5%) (S2 Table).

**Table 1:** Demographic characteristics and clinical, neurodevelopmental, and laboratory outcomes of children with CZS-associated microcephaly.

Characteristics	Children with CZS- associated microcephaly	
	No of participants	n (%) or median (IQR)
**Male, n (%)**	42	19 (45.2)
**Preterm, n (%)**	42	4 (9.5)
**CT scan abnormalities, n (%)**	39	39 (100.0)
**Postneonatal epilepsy, n (%)**	28	19 (67.9)
**Anthropometric growth**		
Age in months, median (IQR)	42	28.2 (23.9–32.9)
Length score Z, median (IQR)	42	-1.5 (-3.3 –-0.5)
Weight score Z, median (IQR)	42	-1.3 (-3.1–0.2)
HC score Z, median (IQR)^a^	42	-6.4 (-7.5 –-3.9)
**Neurological evaluation**		
Age in months, median (IQR))	35	27.8 (25.1–31.6)
HINE neurologic scale, median (IQR)	35	25.0 (20.5–33.0)
Range score 0–78		
HINE motor scale, median (IQR)	35	2.0 (1.0–4.0)
Range score 0–26		
HINE behavior scale, median (IQR)	35	13.0 (11.3–15.0)
Range score 3–15		
**Neurodevelopmental Evaluation**		
Bayley-III summary^b^		
Age in months, median (IQR)	33	31.7 (29.4–37.5)
Cognitive scale percentile, median (IQR)	33	0.1 (0.1–0.1)
Language scale percentile, median (IQR)	33	0.05 (0.05–0.05)
Motor scale percentile, median (IQR)	33	0.05 (0.05–0.05)
Corrected age according Bayley raw scores		
Cognitive corrected age in months + days, median (IQR)	33	2.3 (1.3–4.0)
Receptive language corrected age in months + days, median (IQR)	33	4.3 (2.3–7.0)
Expressive language corrected age in months + days, median (IQR)	33	2.6 (2.0–5.0)
Fine motor corrected age in months + days, median (IQR)	33	0.6 (0.5–1.6)
Gross motor corrected age in months + days, median (IQR)	33	1.6 (0.5–3.0)
**Ophthalmological evaluation**		
Age in months, median (IQR)	28	29.2 (28.1–32.3)
Inadequate visual function, n (%)	28	25 (89.3)
Abnormalities at funduscopic eye exam, n (%)	26	15 (57.7)
**Auditory evaluation**		
Age in months, median (IQR)	34	27.9 (25.7–30.5)
Abnormal BERA	22	4(18.2)
Abnormal OEA	18	3(16.7)
Auditory response, n (%)		
No response	32	3 (9.4)
Doubtful reaction to stimuli or asymmetry of response	32	8 (25.0)
Reacts to stimuli from both sides	32	21 (65.6)
Absence of cochlear-palpebral reflex, n (%)	34	4 (11.8)
Abnormalities at Auditory behavior tests, n (%)	34	25 (73.5)
Abnormal Auditory development, n (%)	34	29 (85.3)
ZIKV laboratory result		
PRNT positive at birth or during the follow up	42	37 (88.1)
PRNT positive result during pregnancy obtained at an outside laboratory not through the study	42	3 (7.4)
Unavailable	42	2 (4.5)

CT: computerized tomography; ZIKV: Zika virus; PRNT: Plaque reduction neutralization test; HINE: Hammersmith Infant Neurological Examination; Bayley-III: Bayley Scales of Infant and Toddler 3rd edition; TEOEA; transient evoked otoacoustic emissions BERA: brainstem electric response audiometry.

^a^ Two children had a HC > -2 SD.

^b^ Only one child had reached composite scores corresponding to mild delay (71 to 85 [-2 SD to -1 SD]) in the all-Bailey’s Bayley’s scales.

The number of participants reflects the availability of specific information.

Conversion factors: 1 month = 30.417 days and 0.1 months = 3.0417 days.

**Fig 1:**
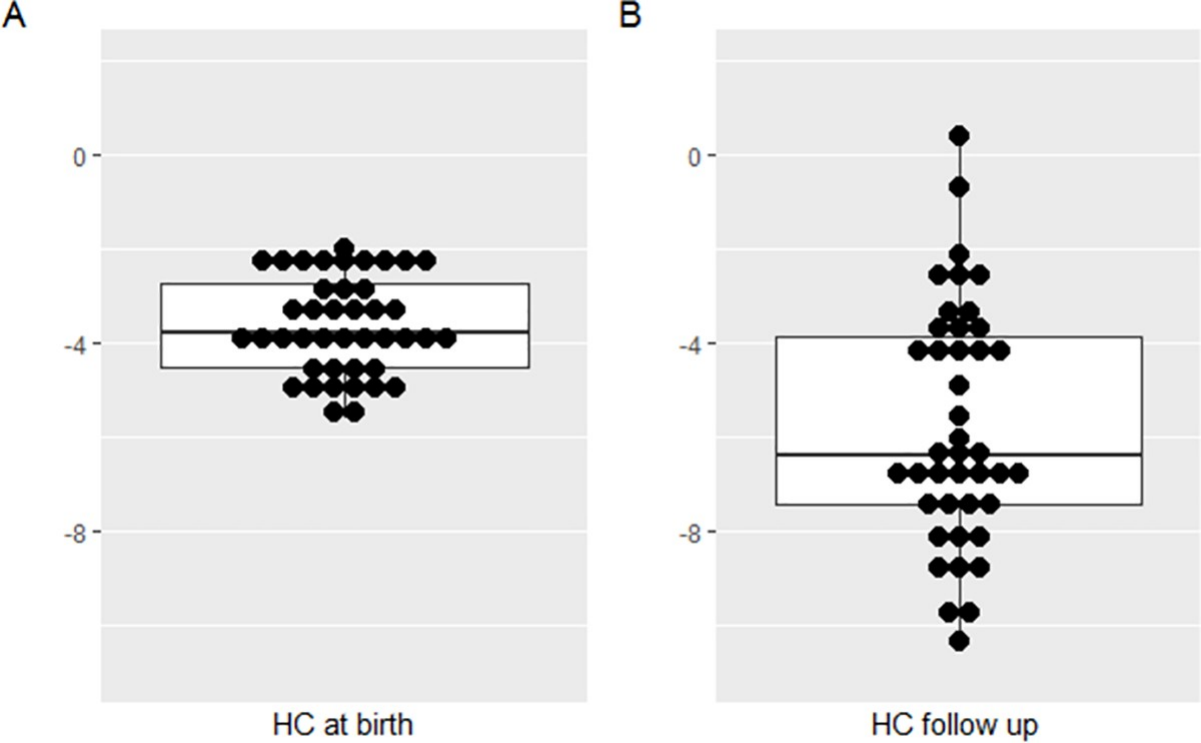
Head circumference of children with Congenital Zika Syndrome-associated microcephaly. Comparison of A) Z-score at birth (INTERGROWTH-21 parameters and B) Z-score at follow up (WHO parameters). The two children with HC Z-score >-2 SD at follow up both had a history of hydrocephalus.

The median age at anthropometric follow up evaluation was 28.2 months (IQR 23.9–32.9). At follow up, two children with a previous history of hydrocephalus had a HC Z-score between 0 and -2. Nineteen (45.2%) had a length Z-score less than -2, 18 (42.9%) had a weight Z-score less than -2, and 36 (85.7%) had a HC Z-score less than -3 (considered severe microcephaly) (Table 1 and Fig 1). When we compared HC at birth [Z score of -3.8(IQR -4.5 –-2.7)] with HC at the follow-up [Z score of -6.4(IQR -7.4 –-3.9)], we found a significant decrease (p < 0.001) in HC (Fig 1).

**Fig 2:**
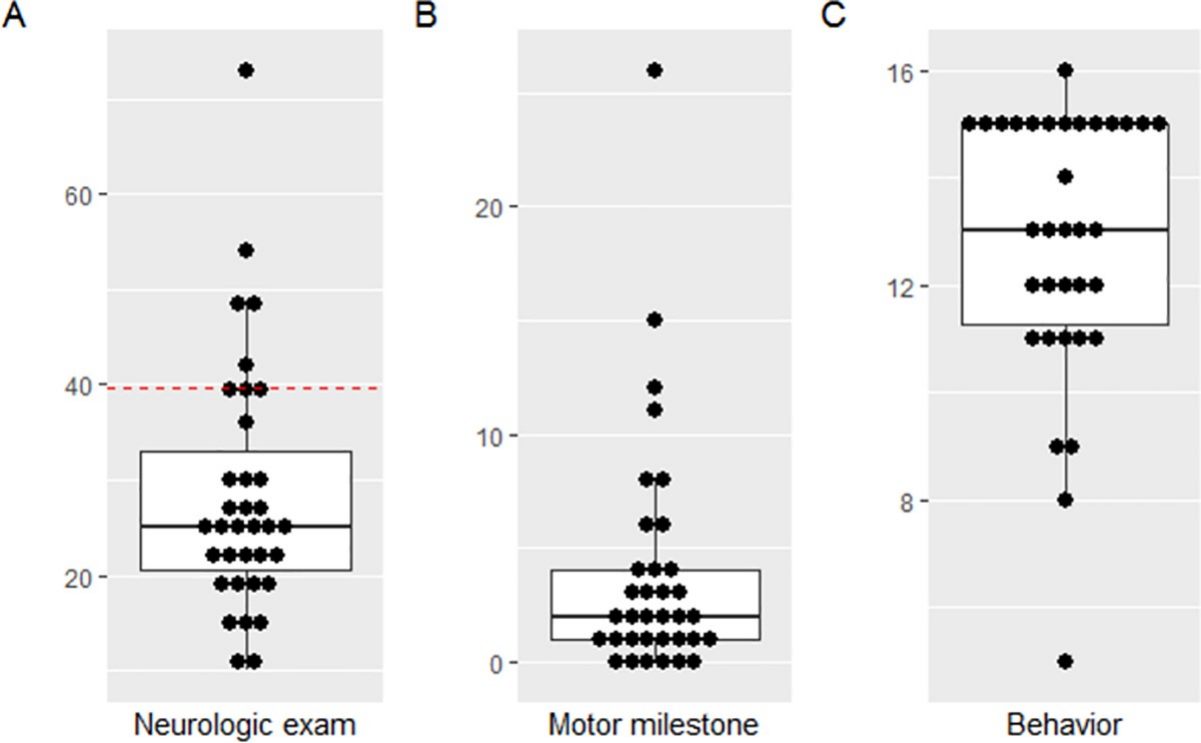
Hammersmith Infant Neurological Examination scores for children with Congenital Zika Syndrome associated microcephaly. A) The main section–neurological exam; B) The second section–motor milestone; and C) The third section—child behavior.

All children had cerebral palsy, bilateral spasticity that compromised their movement, poor head control and hyperreflexia. This required all children to be transported in a manual wheelchair in all settings. All children were categorized by a neuropediatrician as Level V according to the Gross Motor Function Classification System. Among 35/42 (83.3%) children with HINE results, median age was 27.8 months (IQR 25.1–31.6). All children had low scores in the HINE neurological section and significant delays in achievement of motor milestones. The median HINE neurological section score was 25.0 (20.5–33.0) and median motor score was 2.0 (IQR 1.0–4.0). The mean score for the HINE behavioral section was much better at 13.0 (IQR 11.3–15.0) (Table 1 and Fig 2).

**Fig 3:**
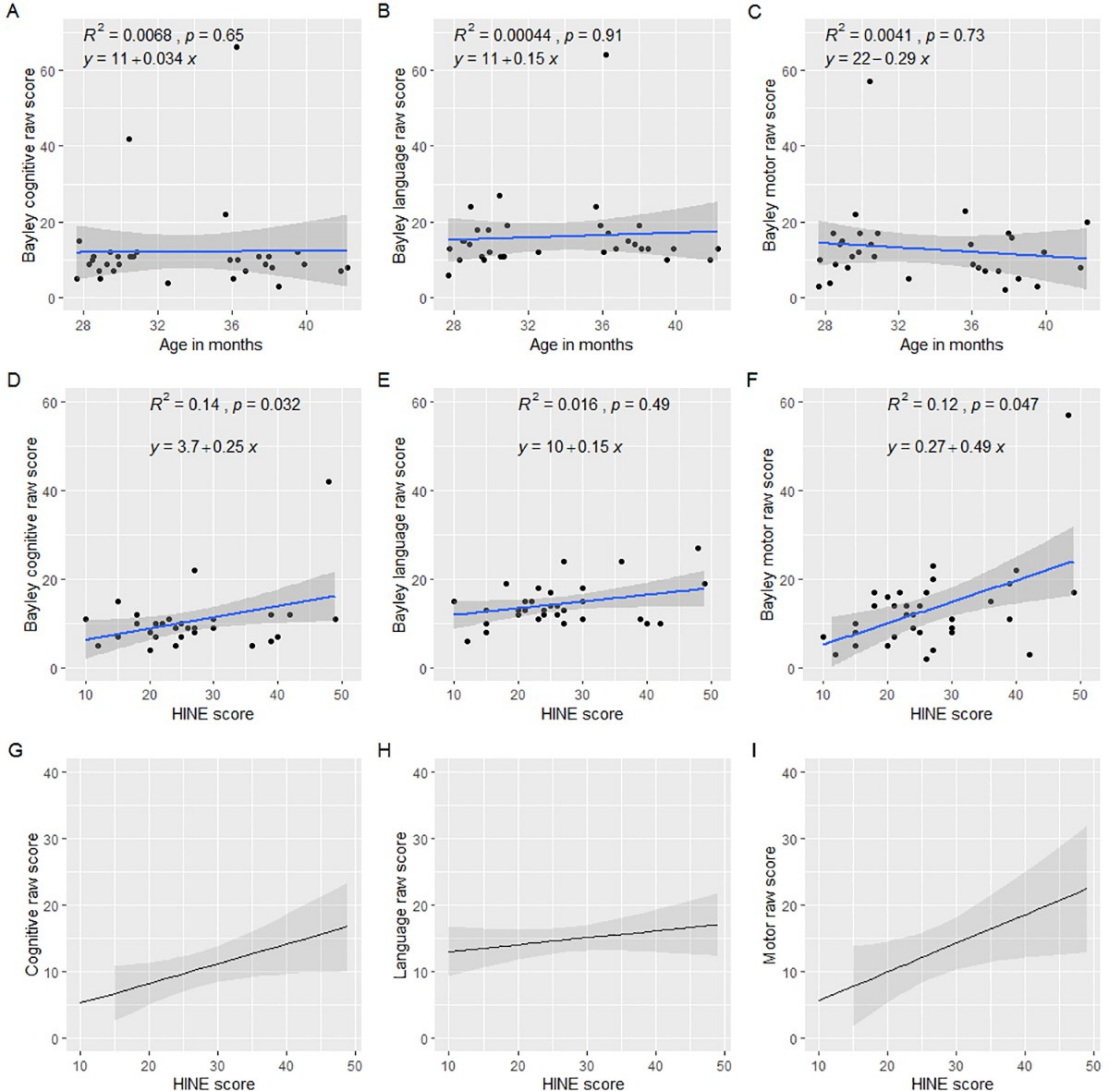
Neurodevelopment of children with Congenital Zika Syndrome-associated microcephaly. Raw Bayley scores over time (A, B, and C). Correlation of raw Bayley scores with the HINE neurological assessment (first section) score (D, E, and F). Linear regression model of the association between HINE score and Bayley raws scores adjusted by prematurity, child age and head circumference (G, H, and I).

For the 33/42 (78.5%) children with a complete Bayley-III, median age was 31.7 months (IQR 29.4–37.5). In our evaluation of child development using Bayley scales, 32/33 (97.0%) had a severe developmental delay in all Bayley-III scales while 1/32 (3%) had only a mild delay in all Bayley-III scale evaluated (Table 1). Composite and raw scores did not show any trend when compared with the age at follow up (Fig 3).

When we compared developmental outcomes based on Bayley scales with the neurological manifestations summarized in the HINE neurological section, HINE scores were positively correlated with the Bayley cognitive (R^2^ = 0.14; p = 0.032) and motor (R^2^ = 0.12; p = 0.047) domains (Fig 3). Furthermore, head circumference Z-scores also were correlated with motor domain (R^2^ = 0.30; p = 0.005) (S1 Fig). In total, the linear regression included 28/42 (66.7%) children. In this regression, we found that better neurological HINE scores were associated with better cognitive raw scores (β = 0.29; CI 95% = 0.02–0.57) and with improved motor raw scores (β = 0.43; CI 95% = 0.04–0.82) after adjusting for head circumference, prematurity and age at neurodevelopmental evaluation. Furthermore, we found that greater head circumference at follow up was associated with higher cognitive and motor raw scores (β = 1.27; CI 95% = 0.01–2.53) and (β = 2.03; CI 95% = 0.25–3.81) (Table 2 and Fig 3). Ophthalmological and auditory findings are summarized in Table 1 and CT scan findings are summarized in S2 Table.

**Table 2:** Linear regression, factors associated with Bayley-III raw scores in children with microcephaly associated with the Congenital Zika Syndrome.

Factors	Bayley cognitive scale raw score			Bayley language scale raw score			Bayley motor scale raw score		
	β	CI	p	β	CI	P	β	CI	p
HINE neurological score	0.29	0.02 – 0.57	**0.035**	0.10	-0.10 – 0.31	0.311	0.43	0.04 – 0.82	**0.030**
Age at time of Bayley exam	-0.11	-0.68 – 0.45	0.681	-0.13	-0.56 – 0.29	0.526	-0.31	-1.11 – 0.49	0.427
Preterm	2.04	-6.05 – 10.14	0.607	0.79	-5.33 – 6.90	0.792	2.80	-8.65 – 14.25	0.618
HC Z-score	1.27	0.01 – 2.53	**0.048**	0.46	-0.49 – 1.41	0.326	2.03	0.25 – 3.81	**0.027**

β: standardized (regression) coefficients; HINE: Hammersmith Infant Neurological Examination.

## Discussion

This is one of the few studies following children with CZS-associated microcephaly beyond two years of age. As expected, these children demonstrate severe language, cognitive, and motor delays as measured by the Bayley-III neurological exam, as well as severe neurological symptoms including bilateral spasticity that compromises their movement, posture, and balance. Among these children, however, there are important differences in their neurological and neurodevelopmental profiles that need to be understood.

Although widely used tools like the composite scores from the Bayley III exam are effective ways to characterize most children [14], we found that they have difficulties differentiating between children with CZS-associated microcephaly, due to the severity of developmental delays within this population [5]. Furthermore, as these children grow, and their gap relative to the standard reference groups for their age used by these tests increases, this effect may worsen. Thus, in order to effectively characterize differences among these children, alternative approaches may be needed.

To that end, we found that HINE scores and Bayley-III raw scores were able to capture the heterogeneity of neurological capabilities among children with CZS-associated microcephaly. Furthermore, we observed that HINE scores could be predictive of cognitive and motor developmental progress at the time of follow-up. A few studies have previously used HINE to evaluate infants with CZS-associated microcephaly, however ours is the first to associate HINE scores with the cognitive and motor development of these children [5, 7]. This is important because HINE is an early neurological examination tool, which is short and easy to perform, and which unlike Bayley does not require specific materials. Furthermore, HINE scores are already used to evaluate developmental and functional prognoses for other developmentally impaired children, such as those with cerebral palsy [13, 17]. While we found that composite scores from Bayley scales were useful in evaluating children without microcephaly and helped to understand the trends of their neurodevelopment [15, 16, 18], children with CZS-associated microcephaly often had severe neurological alterations, including neurosensory impairment which complicated performing an evaluation as active as Bayley-III [2, 3, 7, 19–21].

In a 2011 paper, Jary et al. discuss the importance of using alternatives to the Bayley-II exam composite scores when evaluating children with severe impairments [10]. In particular, they highlight the ability of Bayley raw scores to demonstrate the heterogeneous development of severely developmentally delayed children. This effect held true in our cohort when using the more recent edition of the Bayley exam, Bayley III. In our study, all children were around the 0.05th percentile, and raw score analysis was necessary to observe the differences between children that was present in their HINE scores.

Head circumference is another easy measurement that we found was associated with neurodevelopment. In a study in Salvador of children with CZS-associated microcephaly, it was described that between follow ups at one and two years after birth, there was a positive correlation between HC and both cognitive and motor performance [5, 6]. In our study, when focusing only on children with HC more than 2SD below average and excluding one child with only mild global delay, we found that HC and HINE scores were positively correlated with cognitive and motor development. This underscores the value of following a simple protocol which includes both these measurements during evaluations of children born with potentially CZS-associated neurological impairments.

It is important to note that while reduced HC is a useful marker of potential neurological alterations due to CZS, children born with a normal head circumference should still undergo further evaluation [3]. Notably, in our study, two children with both a diagnosis of microcephaly and hydrocephaly had a normal head circumference at the time of follow up, and were not included in the head circumference analysis. This is consistent with previous work by Pereira H. et al., which describes nineteen children with a normal head circumference at birth and neuroimaging findings typical for CZS patients, of which fifteen developed postnatal microcephaly and only four maintained normal head circumference through the second year of life [3]. Pereira H et al. concluded that this phenomenon could be due to the total or partial resolution of ventriculomegaly [3]. It is likely that the two children with normal head circumference at follow up in our study will continue to develop slowly.

Our study has some relevant limitations. First, the number of participants in our cohort was small, which limited the study’s statistical power. That said, this study represents a unique opportunity to understand a rare event in the form of microcephaly, which is a severe consequence of congenital Zika virus infection. Second, despite multiple attempts, it was difficult to complete all clinical assessments for all children, which may have affected the rate of defects identified in our study. This is primarily because the extensive number of assessments involved deterred some mothers and their children from persisting with evaluations. In order to combat this, the research team often provided families with transportation or performed home visits for these evaluations, however some assessments could not be performed outside of the hospital.

Another limitation of our study is the use of HINE exams in children older than 24 months. Previous studies had identified neurodevelopmental delays of approximately 20 months in children with CZS-associated microcephaly by age two, indicating that the HINE exam might still be an appropriate tool to use when evaluating them. This is consistent with our findings from the Bayley-III evaluations in which all children scored below the 0.05th percentile, corresponding to an adjusted relative age between 20 days to 4 months. A similar age correction was used by Lind A. et al. for preterm children (gestational age between 23–35 weeks) [22]. They performed HINE exams based on a corrected age of two years, equivalent to a time of 26 to 29 months after birth [22]. Furthermore, a study by Nielsen-Saines K. et al. performed HINE exams on children with previous intrauterine ZIKV exposure including children with CSZ-associated microcephaly aged 7 to 32-months, indicating that it can still be used for older children [7]. Another reason to use the HINE exam is that, unlike the Bayley III exam, it does not require extensive tools or materials to perform, making it practical in resource-poor settings, as in our study site.

We were not able to relate the link between HINE and Bayley results to imaging results because CT scans were only available for the first year of life. Additionally, it would have been ideal to also perform MRIs in order to better identify migrational defects. Finally, we were limited to use of a cross-sectional study design which makes it difficult to establish a firmly causal association between the factors examined and the outcomes we explore in our study. Thus our findings are correlational instead, and further prospective studies that follow children with CZS-associated microcephaly during the growth are necessary.

## Conclusions

We found that children with CZS-associated microcephaly experience major neurodevelopmental delays and severe neurological outcomes including spasticity which compromises their movement, posture, and balance. Still, there is significant variation among these children and they demonstrate heterogeneous development patterns. In order to better understand the differences between these children and to identify early interventions which will reduce the disease burden, it is first necessary to develop either new evaluative tools or standardized adjustments to existing ones, which reflect this heterogeneity and can be used to follow and evaluate the progression of the disease. Health professionals need easy, practical, and reliable tests which can predict the longer term outcomes of their patients, and can help them to design plans for their therapy and treatment.

## Supporting information

S1 Data Excel spreadsheet containing, in separate spreadsheets, the data used in tables and figures. (XLSX)

S1 Table Mother characteristics. (DOCX)

S2 Table Cranial computed tomography scans (CT) of children with Congenital Zika Syndrome associated microcephaly. (DOCX)

S1 Fig Head circumference of children with CZS associated microcephaly associated with neurodevelopmental and neurological outcomes. A) Cognitive Bayley III scale raw score, B) Language Bayley III scale raw score C) Motor Bayley III raw score and D) Hine neurological section score.
(TIF)
