# Supplementary material for: Heterogeneous development of children with Congenital Zika Syndrome-associated microcephaly
Source: PLoS One. 2021 Sep 15;16(9):e0256444. doi: 10.1371/journal.pone.0256444 (PMC8443077; doi:10.1371/journal.pone.0256444)
Supplement: S1 Table — (DOCX) [file pone.0256444.s002.docx]

S1Table. Mother Characteristics

| **Characteristics** | **Total**  **No. (%) or median (IQR)**  **(N=42)** | **Children born at**  **HGRS**  **No. (%) or median (Range)**  **(N=29)** | **Children born**  **outside HGRS**  **No. (%) or median (Range)**  **(N=13)** |
| --- | --- | --- | --- |
| Median mother age, years | 25/42 (15 – 42) | 25/29 (18 – 39) | 26/13 (15 – 32) |
| Black ethnicity^1^ | 20/37 (54.1) | 14/28 (50%) | 6/9 (66.6) |
| Lower educational level^1^ | 11/36 (30.6) | 8/27 (29.6) | 3/9 (33.3) |
| Dissatisfaction with family income | 22/30 (73.3) | 14/21 (66.6) | 8/9 (88.9) |
| Concern about lack of food | 21/30 (70.0) | 16/21 (76.2) | 5/9 (55.6) |
| Food restriction or reduction due to lack of money | 19/30 (63.6) | 15/21 (71.4) | 4/9 (44.4) |
| Poor self-perceived health | 5/30 (13.3) | 4/21 (19.0) | 0/9 (0%) |
| **Medical history** |  |  |  |
| Pre-existing diseases | 6/37 (16.2) | 6/28 (21.4) | 0/9 (0%) |
| Prior sexually transmitted infection | 1/37 (2.7) | 0/28 (0) | 1/9 (11.1) |
| TORCH infections | 0/42 (0) | 0/28 (0) | 0/13 (0) |
| **Symptoms during pregnancy** |  |  |  |
| Rash | 17/38 (44.7) | 14/28 (50.0) | 3/10 (30.0) |
| Fever | 17/38 (44.7) | 14/28 (50.0) | 3/10 (30.0) |
| Conjunctival injection | 10/38 (26.3) | 6/28 (21.4) | 4/10 (40.0) |
| Myalgia | 19/38 (50.0) | 15/28 (53.6) | 4/10 (40.0) |
| Arthralgia | 11/38 (28.9) | 7/28 (25.0) | 4/10 (40.0) |

‡ Black ethnicity was compared with White and Mixed ethnicities. Lower educational level was defined as no education or only up to first or second grade completed
