# Supplementary material for: Heterogeneous development of children with Congenital Zika Syndrome-associated microcephaly
Source: PLoS One. 2021 Sep 15;16(9):e0256444. doi: 10.1371/journal.pone.0256444 (PMC8443077; doi:10.1371/journal.pone.0256444)
Supplement: S2 Table — (DOCX) [file pone.0256444.s003.docx]

**S2 Table. Cranial computed tomography scans (CT) of children with Congenital Zika Syndrome-associated microcephaly**

|  | **Total**  **N = 39**  **n (%)** | **Children born at HGRS**  **N = 28**  **n (%)** | **Children born outside HGRS**  **N = 11**  **n(%)** |
| --- | --- | --- | --- |
| Calcifications present: |  |  |  |
| Parenchymal | 33 ( 84.6) | 24 ( 85.7) | 9 ( 81.8) |
| Subcortical | 30 ( 76.9) | 21 ( 75.0) | 9 ( 81.8) |
| Periventricular | 15 ( 38.5) | 8 ( 28.6) | 7 ( 63.6) |
| Basal ganglia | 23 ( 59.0) | 16 ( 57.1) | 7 ( 63.6) |
| Stippled pattern | 28 ( 71.8) | 21 ( 75.0) | 7 ( 63.6) |
| Laminar pattern | 12 ( 30.8) | 10 ( 35.7) | 2 ( 18.2) |
| Ventriculomegaly | 34 ( 87.2) | 24 ( 85.7) | 10 ( 90.9) |
| Simplified gyral pattern | 31 ( 79.5) | 23 ( 82.1) | 8 ( 72.7) |
| Reduced cerebellar volume | 12 ( 30.8) | 8 ( 28.6) | 4 ( 36.4) |
| Corpus callosum agenesis/dysgenesis | 28 ( 71.8) | 21 ( 75.0) | 7 ( 63.6) |
| Agenesis | 5 ( 12.8) | 4 ( 14.3) | 1 ( 9.1) |
| Dysgenesis | 22 ( 56.4) | 17 ( 60.7) | 5 ( 45.5) |
| Cerebral atrophy | 27 ( 69.2) | 22 ( 78.6) | 5 ( 45.5) |
| Thalamic calcifications | 16 ( 41.0) | 12 ( 42.9) | 4 ( 36.4) |
| Brainstem calcifications | 12 ( 30.8) | 8 ( 28.6) | 4 ( 36.4) |
| Cerebellar calcifications | 2 ( 5.1) | 2 ( 7.1) | 0 ( 0.0) |
