# Supplementary figures and images for: Heterogeneous development of children with Congenital Zika Syndrome-associated microcephaly
Source: PLoS One. 2021 Sep 15;16(9):e0256444. doi: 10.1371/journal.pone.0256444 (PMC8443077; doi:10.1371/journal.pone.0256444)

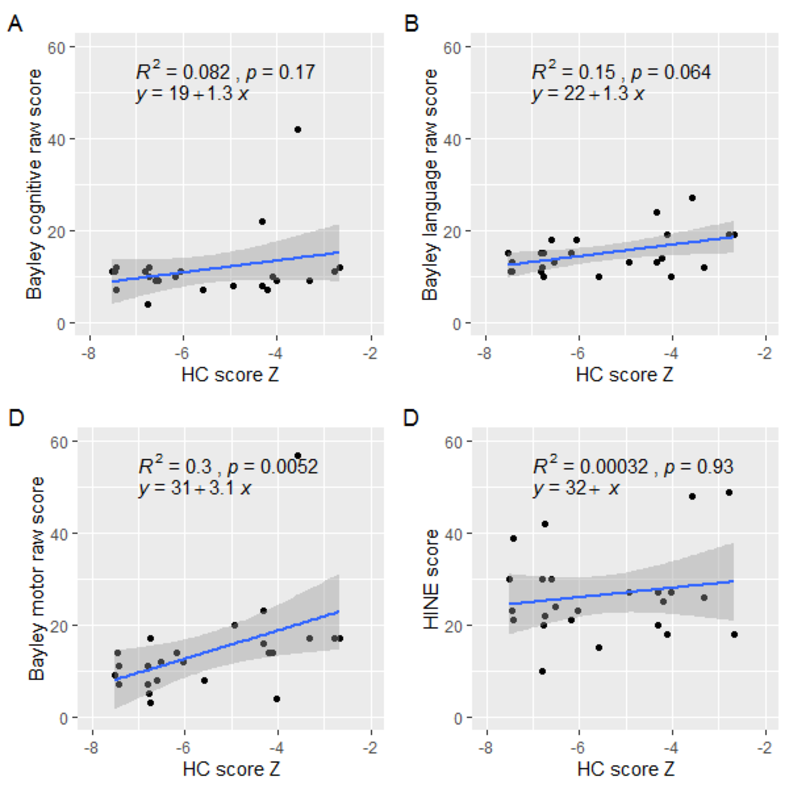

Supplement: S1 Fig — A) Cognitive Bayley III scale raw score, B) Language Bayley III scale raw score C) Motor Bayley III raw score and D) Hine neurological section score. (TIF) [file pone.0256444.s004.tif]
